# Synergistic Effects of 1-MCP Fumigation and ε-Poly-L-Lysine Treatments on Delaying Softening and Enhancing Disease Resistance of Flat Peach Fruit

**DOI:** 10.3390/foods12193683

**Published:** 2023-10-07

**Authors:** Yanli Zheng, Xiaoyu Jia, Lihua Duan, Xihong Li, Zhiyong Zhao

**Affiliations:** 1 State Key Laboratory of Food Nutrition and Safety, College of Food Science and Engineering, Tianjin University of Science and Technology, Tianjin 300457, China; zhengyanli3344@163.com (Y.Z.); duanlihuaok@126.com (L.D.); lixihong606@outlook.com (X.L.); 2 Institute of Agricultural Products Preservation and Processing Science and Technology, Tianjin Academy of Agricultural Sciences, Tianjin 300384, China; 3 Instiute of Agro-Products Processing Science and Technology, Xinjiang Academy of Agricultural and Reclamation Science, Shihezi 832000, China

**Keywords:** flat peach, 1-MCP, ε-PL, softening, cell wall metabolism, reactive oxygen species, disease resistance

## Abstract

Flat peach, a predominant fruit consumed in China, is highly susceptible to softening and perishable. The impact of 1-methylcycloproene (1-MCP) fumigation combined with ε-poly-L-lysine (ε-PL) on softening and postharvest reactive oxygen species (ROS) and phenylpropanoid pathway metabolisms in peaches and its relationship to disease resistance were investigated. Findings revealed that a combination of 1 µL L^−1^ 1-MCP and 300 mg L^−1^ ε-PL effectively suppressed the activity of cell-wall-degrading enzymes and the disassembly of cell wall structure, thus maintaining higher firmness and lower decay incidence. Compared to the control group, the synergistic approach bolstered enzymatic responses linked to disease resistance and ROS-scavenge system, consistently preserving total phenolics, flavonoids, ascorbic acid, and glutathione levels. Concurrently, the accumulation of hydrogen peroxide and malondialdehyde was significantly diminished post-treatment. These results show that there is good synergistic effect between 1-MCP and ε-PL, which could effectively maintain the quality of flat peach fruit by modulating cell wall metabolism and enhancing the resistance.

## 1. Introduction

Flat peach (*Prunus persica* L. Batsch. var. *Compressa* Bean), a variant of the peach (*Prunus persica* (L.) Batsch), belongs to the Rosaceae family [1]. Its cultivation thrives primarily in regions such as Beijing, Xinjiang, and Zhejiang, China [2]. Esteemed for its rich nutritional and functional components, flat peach garners considerable acclaim among consumers [3]. However, its postharvest respiratory climacteric nature, coupled with a heightened ethylene production rate, renders it highly perishable [4]. Susceptibility to adverse storage and transport conditions—manifested as mechanical injuries, accelerated softening, moisture loss, physiological decay, and microbial contamination—curtails its shelf life considerably [5]. Thus, strategies to mitigate postharvest physiological degradation and disease onset are imperative to prolonging fruit longevity.

Currently, cold storage stands as the predominant strategy to retard postharvest peach softening, yet its utilization risks inducing chilling injuries. Consequently, it often synergizes with alternative treatments [6]. Empirical investigations underscore the efficacy of 1-MCP in safeguarding postharvest physiological integrity in fruits and vegetables [7]. Treatment with 1-MCP can delay fruit softening by inhibiting the activity of enzymes related to cell wall degradation, including pectate lyase (PL), pectin methylesterase (PME), polygalacturonase (PG), and *β*-galactosidase (*β*-Gal) and in turn can help preserve cell wall components, such as cellulose, pectin, and hemicellulose [8,9]. However, 1-MCP is deficient in postharvest fruit decay control and shelf quality maintenance and often needs to be combined with other treatments [10,11]. Treated with 100 nLL^−1^ of 1-MCP, the decay rate of strawberries and oranges increased [12,13]. Moreover, the sensitivity of grapes to *Penicillium digitatum* did not change after 1-MCP fumigation [14]. On the other hand, the application of 1-MCP, while maintaining the hardness of the plums, also reduced anthocyanin biosynthesis during storage [15]. Thus, scouting for complementary 1-MCP regimens, capable of both decelerating fruit softening and thwarting postharvest decay, emerges as a research priority.

ε-Poly-L-lysine (ε-PL) is recognized as an innate antagonist against plant pathogens [16]. Its broad-spectrum antibacterial properties have led to its widespread application in the food industry [17,18]. Notably, ε-PL proficiently mitigates the proliferation of both Gram-positive and Gram-negative bacteria as well as fungi. Its mode of action encompasses electrostatic adherence to cell membranes, modulating membrane permeability, disrupting cellular structures, and constraining cellular metabolism [19,20]. Presently, ε-PL finds utility in the preservation of diverse horticultural products including apples, bamboo shoots, kiwifruits, carrots, and citrus [16,21,22,23]. Research indicates that the amalgamation of ε-PL with alternate techniques fortifies the postharvest quality of fruits and vegetables. For instance, ultrasonic waves combined with ε-polylysine enhanced the microbiological and storage attributes of fresh-cut lettuce [24]. Likewise, an integration of ε-polylysine and thermosonication bolstered the longevity of pasteurized milk by undermining cellular architectures [25]. Furthermore, the dual application of ε-polylysine and chitosan coating has been documented in Pacific white shrimp preservation [26].

To date, no literature elucidates the cooperative modulation of 1-MCP and ε-PL in governing postharvest physiology and pathogenic susceptibility in peaches. This investigation pivots on the merger of 1-MCP fumigation and ε-PL atomization for postharvest peach conservation. The ability to maintain fruit quality was studied by analyzing the cell wall metabolism, reactive oxygen species (ROS) metabolism and disease resistance. The aim of this study is to lay the foundation for further application of physiological and disease control preservatives in the postharvest disease control and quality maintenance of fruits.

## 2. Materials and Methods

### 2.1. Peach Materials and Treatments

“Yinggeer” flat peaches (*Prunus persica* L. Batsch. var. *Compressa* Bean) were cultivated in an orchard in Shihezi, Xinjiang, China (44°16′ N, 85°78′ E, Altitude 450.8 m), with harvest executed in August 2021. The mature fruit exhibited the following characteristics: firmness of 14.97 ± 0.84 N, soluble solids content of 12.7 ± 0.53%, and titratable (malic acid) acidity of 0.15 ± 0.006%. Experimental treatment according to the described process: fruit selection → 1-MCP Fumigation → transport → ε-PL soaking → precooling (Including drying) → packaging→ storage at 0 °C. The specific parameters are described below. Only morphologically consistent fruits, devoid of visible defects or ailments, were selected. These peach samples underwent 1-MCP fumigation in a hermetically sealed 1.0 m^3^ polyethylene tent (0.12 mm thickness) at 16 ± 0.5 °C, where a preformulated solution released 1 µL L^−1^ 1-MCP vapor [27]. Controls were similarly ensconced but without any treatment. Post-fumigation, peaches were relayed to a Tianjin agricultural product storage laboratory. Preliminary assays, encompassing ε-PL concentrations of 150, 300, and 450 mg L^−1^ under 0 ± 0.5 °C for a 30-day period, discerned the 300 mg L^−1^ ε-PL concentration as most efficacious against peach decay. The flat peaches, pre-treated with or without 1-MCP, were randomly immersed in ε-PL solution (300 mg L^−1^) (as ε-PL+1-MCP or ε-PL) or distilled water (as 1-MCP or the control) for 5 min. The fruit were placed in a 0 °C cold storage for pre-cooling for 24 h before proceeding with the next step of processing. Once air-dried, fruits were packaged in 0.05 mm polyethylene bags (25 peaches/bag, 30 bags/group) and stored at 0 °C with 90–95% relative humidity for 30 d.

### 2.2. Determination of Firmness and Ethylene Production

The firmness of the fruit was measured on days 0, 6, 12, 18, 24 and 30 using the GY-4 digital firmness tester (Shandong Fangke Instrument Co., Ltd., Weifang, China). This device, fitted with a 3.5 mm-diameter probe, was inserted at three equidistant points around the fruit’s equatorial zone [27]. Firmness readings were expressed in Newtons (N). Ethylene production in samples was assayed via gas chromatography (GC-2014, Japan Shimadzu Corp, Tokyo, Japan) employing the method of Mullins et al. [14], and the result was expressed as μL kg^−1^ h^−1^.

### 2.3. Microscopic Observations

Microstructures of the peach pulp specimens from both initial and 30 d were analyzed with a scanning electron microscope (SU3500, Japan Hitachi Corp, Tokyo, Japan), in alignment with the procedures outlined by Jia et al. [28]. For preparatory measures, peach tissue samples were sized to 1 mm × 3 mm × 3 mm dimensions, swiftly subjected to liquid nitrogen cooling at −196 °C, and subsequently lyophilized at −65 °C. Pre-observation, these specimens underwent a gold sputtering process for 120 s and were subsequently inspected at ×300 magnification, applying an acceleration voltage of 15.0 kV.

### 2.4. Assays of Cell Wall Modifying Enzymes

The activities of PG, *β*-Gal, and cellulase were ascertained using previous methods [29,30]. Specifically, a 10 g peach sample was pulverized with a sodium acetate buffer solution (40 mM, pH 5.2) enriched with polyvinylpyrrolidone (1%, *w*/*v*), 1 M NaCl, and mercaptoethanol (2%, *v*/*v*). The resulting mixture was centrifuged at 10,000× *g* for 30 min, after which the separated supernatants (yielding crude enzyme extracts) were employed for enzymatic activity assessments.

For PG activity, 0.1 mL of the crude enzyme combined with 0.3 mL polygalacturonic acid (1%, *w*/*v*) and 0.2 mL sodium acetate buffer (40 mM, pH 4.0). This solution underwent incubation at 37 °C for 30 min. Following the addition of 1 mL of 3, 5-dinitrosalicylic acid (0.63%, *w*/*v*), the reaction was stopped with a 5 min boiling bath. PG activity’s absorbance was quantified at 540 nm, with D-galacturonic acid as a reference. For *β*-Gal activity, 0.2 mL of the crude enzyme extract combined with 0.5 mL of 50 mM sodium acetate buffer, and 10 mM p-nitrobenzene-*β*-D-Galactoside 0.2 mL. After incubating the solution at 37 °C for 1 h, 2 mL of sodium carbonate (Na_2_CO_3_, 1 M) was introduced. *β*-GAL activity’s absorbance was determined at 400 nm using p-nitrophenol as a standard. For cellulase activity, the mixture consists of 0.1 mL of crude enzyme, 0.5 mL of 0.1 M sodium acetate buffer, and 0.4 mL of 1% carboxymethyl cellulose, with subsequent steps aligning with those for PG activity. PME activity was determined as per Zhao et al. [31]. A 4 g peach sample was pulverized with 8.8% NaCl (*w*/*v*) solution in an ice bath, agitated for 4 h, and centrifuged at 10,000× *g* for 20 min at 4 °C. The supernatant was curated as the enzyme extract. This extract (0.1 mL) was combined with 0.75 mL distilled water, 2 mL pectin solution (Sigma, St. Louis, MO, USA, from citrus fruit), and 0.15 mL bromothymol blue (0.01%, *w*/*v*). PME activity’s absorbance was evaluated at 620 nm, with results indicated as U kg^−1^.

### 2.5. Cell Wall Ultrastructure

Random peach flesh samples, sized 1 mm^3^, were immersed in 2.5% glutaraldehyde for 5 h and rinsed with phosphate buffer (0.1 M, pH = 7.4) at 4 °C for 15 min. Subsequent fixation, dehydration, and embedding steps were conducted as per Wang et al. [32]. Micrographs of initial and 30-day-old tissues were obtained via a transmission electron microscope (Talos F200X, America FEI Corp, Hillsboro, OR, USA).

### 2.6. Assays of Physicochemical Properties

#### 2.6.1. Decay Incidence

Decay rates were measured using a prior visual evaluation technique [28]. Peaches showing surface mycelia development or color change due to skin browning or fungal growth were considered decayed. This evaluation was iterated six times, employing 50 peaches per cycle.

#### 2.6.2. Hydrogen Peroxide (H_2_O_2_) and Malondialdehyde (MDA) Contents

The H_2_O_2_ concentrations were quantified following the protocol of Li et al. [33] and are presented as mmol kg^−1^ fresh weight (FW). For the determination of malondialdehyde (MDA), the thiobarbituric-acid-reactive substance methodology [34] was employed. Peach samples (5.0 g) were homogenized in 10 mL of 10% trichloroacetic acid (TCA, 100 g L^−1^) and centrifuged at 16,000× *g* for 15 min. The assay mixture, comprising 2.0 mL supernatant and 2.0 mL of 0.5% thiobarbituric acid (TBA), underwent a boiling treatment, subsequent cooling, and then centrifugation at 10,000× *g* for 10 min. Absorbance was read at wavelengths of 450, 532, and 600 nm. MDA concentrations are denoted as μmol kg^−1^ FW.

#### 2.6.3. Total Phenolics, Total Flavonoids, Ascorbic Acid (AsA), and Glutathione (GSH) Contents

The quantification of total phenolics in nectarines was executed using the previous methodology [35]. Absorbance readings of the samples were acquired using a UV 3600 Plus spectrophotometer (Shimadzu, Kyoto, Japan) at 760 nm. Phenolic content is represented as mg gallic acid equivalents per kg^−1^ FW.

For flavonoid content determination, an adapted protocol from Toor and Savage [36] was followed. A 2.0 g tissue sample was homogenized using 60% ethanol and centrifuged at 12,000 rpm for 20 min at 4 °C; 2 mL of extract, 1 mL of ethanol, 1 mL of 3% AlCl_3_, and 2 mL of sodium acetate buffer (pH 5.5) were mixed evenly. The optical density was measured at 510 nm, and findings are presented as mg rutin equivalents per kg^−1^ FW.

The ascorbic acid (AsA) content was quantified using a sample size of three replicates, with each replicate consisting of five fruits. Assays utilized the 2,6-dichlorophenol-indophenol dye titration technique as delineated previously [37]. Results are presented as mg kg^−1^ FW.

Lastly, the determination of total GSH content was performed according to the Castillo and Greppin protocol [38]. An extract (0.5 mL) was combined with 1.8 mL of 0.15 M PBS (pH 7.8) and 0.2 mL DTNB, incubated at 30 °C for 60 min. Absorbance readings were taken at 412 nm, and the result is expressed in mg kg^−1^ FW.

### 2.7. Assays of Ascorbate Peroxidase (APX), Glutathione Reductase (GR), Peroxidase (POD), Catalase (CAT), and Superoxide Dismutase (SOD) Activities

APX and GR enzymatic activities were quantified employing a refined method from Ma et al. [39]. The APX reaction mixture encompassed 800 µL AsA (3.0 mmol L^−1^), 2.0 mL phosphate buffers (100 mmol L^−1^), 0.5 mL 0.5 mmol L^−1^ H_2_O_2_, 0.2 mL enzyme extract, and 0.5 mmol L^−1^ EDTA. For GR, the mixture contained 3.0 mL of 100 mmol L^−1^ phosphate buffers (pH 7.5), 100 μL of 5.0 mmol L^−1^ oxidized glutathione, 0.2 mL enzyme extract, and 30 μL of 300 μmol L^−1^ NADPH. Optical densities for APX and GR mixtures were noted at 290 nm and 340 nm, respectively, with activities denoted in U g^−1^ FW. The activities of POD, CAT, and SOD were determined with the respective kits. Specifically, a 0.1 g flat peach sample was processed in 1 mL extraction buffer, centrifuged at 10,000× *g*, 4 °C for 10 min, and the resultant supernatant was isolated. Following the kit protocols, reagents were incorporated in sequence, with absorbance readings at 470 nm, 240 nm, and 560 nm post specific reaction intervals. Activity measurements were uniformly represented in U g^−1^ FW.

### 2.8. Assays of Phenylalanine Ammonia-Lyase (PAL), Cinnamate-4-Hydroxylase (C4H), and 4-Coumarate Coenzyme A Ligase (4CL) Activities

The enzyme activity of PAL and 4CL were determined employing Zhang et al.’s method [40]. Absorbance for PAL and 4CL were noted at 290 nm and 333 nm, respectively. The C4H enzymatic activity was gauged using the specific C4H Detection Kit sourced from Shanghai Xige Biotechnology Co., Ltd., Shanghai, China, with absorbance captured at 340 nm. A single unit (U) of C4H activity was characterized by the milligram of protein yielding 1 nmol of NADPH every minute.

### 2.9. Statistical Analysis

Three experiments were conducted systematically for each treatment. Data analysis utilized SPSS 22.0. A one-way ANOVA assessed the data with post hoc comparisons using Duncan’s multiple range tests, significant at *p* = 0.05. Data are reported as mean ± standard deviation.

## 3. Results

### 3.1. Analysis of Appearance Changes, Firmness, Ethylene Production, and Microstructure of Flat Peach Fruit

**Figure 1:**
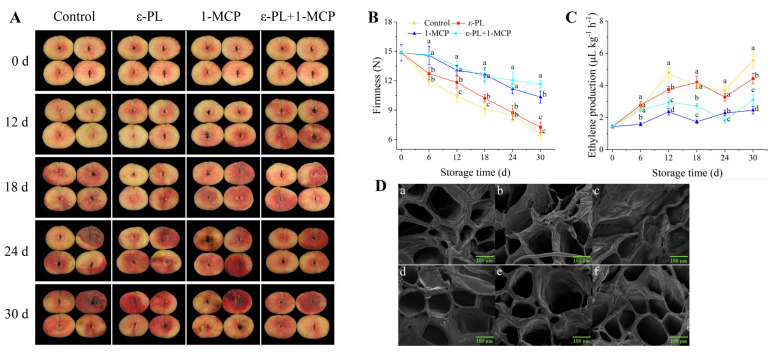
Appearance alterations (**A**), firmness (**B**), and ethylene production (**C**) in flat peach during preservation. SEM illustrations depict the pulp morphology effects of treatments (**D**) throughout storage: (a,b) untreated peach pulp on day 0, (c) control fruit, (d) ε-PL−treated fruit, (e) 1-MCP−treated fruit, and (f) ε-PL+1-MCP−treated fruit on day 30. Results are expressed as mean ± standard deviations, *n* = 3. Different letters show statistically significant differences at *p* < 0.05.

Figure 1A indicates that the appearance quality of flat peach treated with ε-PL+1-MCP was surpassed that of those with control, ε-PL, and 1-MCP groups during the whole storage period. Fruit rot onset in the control group was noted on day 18, intensifying by day 24. By contrast, 1-MCP treated peaches exhibited rot after 18 d, whereas the ε-PL and ε-PL+1-MCP showed decay symptoms on day 24. A declining trend in fruit firmness was evident for all treatments with prolonged storage (Figure 1B). The firmness of peaches in the1-MCP and ε-PL+1-MCP treatment groups was significantly better than that in the control group (*p* < 0.05) during the 6–30 d storage period. The firmness in ε-PL group was 13.53% or 12.07% higher than untreated group at 12 d or 18 d, respectively. Furthermore, from 0–24 d, no discernible difference in firmness was found between ε-PL+1-MCP and 1-MCP treated fruits. The ethylene production in peaches from four groups during storage showed a fluctuating tendency (Figure 1C). Ethylene production was reduced by 21.59%, 50.73%, and 38.36% in 12 d stored and by 20.36%, 55.86%, and 44.11% in 30 d stored fruit which were treated with ε-PL, 1-MCP, and ε-PL+1-MCP, respectively, when compared to the control group. Changes in the micromorphology of the flat peach flesh were observed by SEM (Figure 1D). In the case of cell contour, the structure of pulp was composed of parenchyma cells, showing evident angular and thick cell walls on day 0 (a,b). After 30 d of storage, the tissue structure of control and ε-PL groups was severely damaged, collapsed, and deformed (c,d). Conversely, 1-MCP and ε-PL+1-MCP groups preserved the structure integrity of flat peach tissues after storage lasting 30 d (e,f).

### 3.2. Analysis of Cell-Wall-Degrading Enzymes in Flat Peach Fruit

**Figure 2:**
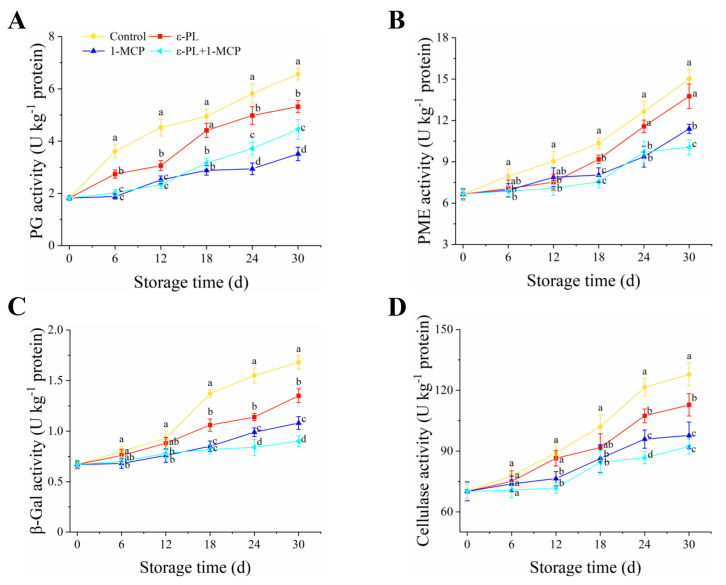
Enzymatic activity trends in flat peach fruit post ε-PL, 1-MCP, and combined ε-PL+1-MCP applications, preserved at 0 °C for 30 days: PG (**A**), PME (**B**), *β*-Gal (**C**) and cellulase (**D**). Results are expressed as mean ± standard deviations, *n* = 3. Different letters show statistically significant differences at *p* < 0.05.

The PG activities of flat peaches in all treatments increased with storage time (Figure 2A). On day 30 of storage, the PG activities in ε-PL, 1-MCP, and ε-PL+1-MCP showed 1.23-, 1.87- and 1.58-times decreases, respectively, compared to the control group. From 18–30 d, both ε-PL+1-MCP and 1-MCP showcased superior inhibitory effects over ε-PL. As indicated in Figure 2B, the PME activity in flat peach showed upward trends during the entire assay period and was inhibited by varying degrees of 1-MCP or ε-PL+1-MCP. The PME level significantly increased (*p* < 0.05), compared to the control group ε-PL, on the 18th day of storage. Conversely, PME activities on the 30th day were noticeably reduced (*p* < 0.05) in the ε-PL+1-MCP compared to the 1-MCP.

*β*-Gal activity in control flat peaches rose slowly from 0–12 d, rose sharply during storage (12–18 d), and enhanced slowly from 18–30 d (Figure 2C). However, *β*-Gal activity in the 1-MCP group and ε-PL+1-MCP group rose slowly. Except for the control group, the *β*-Gal activity significant decreased (*p* < 0.05) between 18 to 30 d of storage. In addition, from 24 d to 30 d, ε-PL+1-MCP showed significantly lower (*p* < 0.05) *β*-Gal activity when compared to ε-PL or 1-MCP alone. Figure 2D showed that cellulase activity in control flat peach increased quickly within the storage 24 d and rose gradually from 24–30 d. Dramatically, from 12–30 d, 1-MCP and ε-PL+1-MCP significantly suppressed a rise in cellulase activity. On the 30th day, cellulase activity in the flat peach treated with ε-PL+1-MCP decreased by 38.61%, 22.32%, and 5.95% compared with the other three treatment groups.

### 3.3. The Ultrastructure of the Cell Wall in Flat Peach Fruit

**Figure 3:**
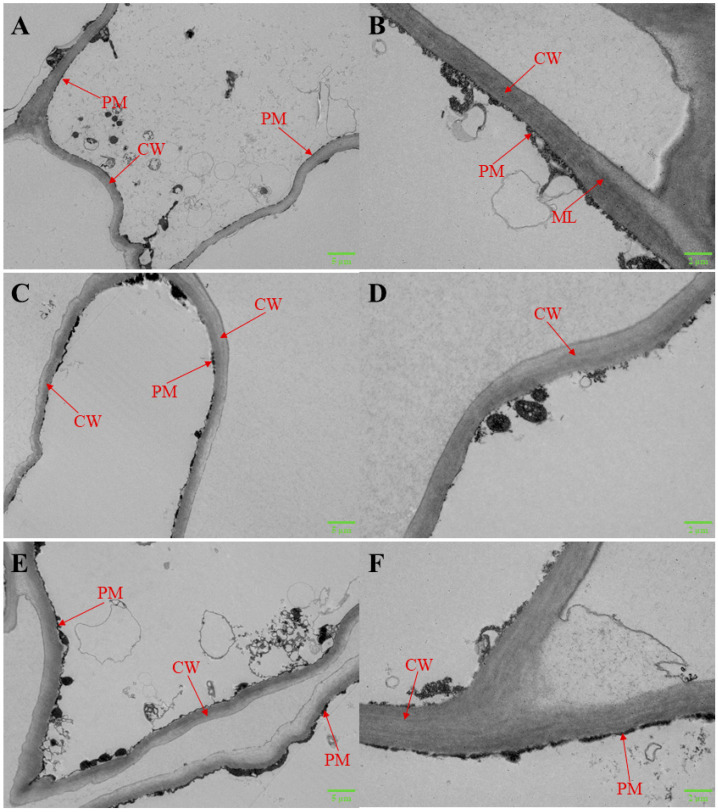
Ultrastructural analysis of pulp tissue from flat peaches using TEM. (**A**,**B**) Control peach on day 0; (**C**,**D**) control peach on day 30 of storage; (**E**,**F**) peach treated with 1-MCP combined with ε-PL on day 30 of storage at 0 °C. CW: cell wall; ML: middle lamella; PM: plasma membrane.

TEM imaging detailed the microstructure of cell walls within flat peach pulp across storage (Figure 3A–F). At inception, the fruit’s plasma membrane adhered closely to an intact cell wall, punctuated by a distinct middle lamella. This wall, within the fruit’s pulp cells, exhibited a continuous fibrous layout (Figure 3A,B). After 30 d of storage, the edge of the cell wall of the control pulp was partly dissolved, and its filaments were swollen and loosened (Figure 3C,D). In contrast, the cell wall was preserved in peach tissues treated with ε-PL+1-MCP (Figure 3E,F). After 30 d of storage, the integrities of the cell wall structure and plasma membrane were maintained in the tissues treated with ε-PL+1-MCP.

### 3.4. Physicochemical Properties of Flat Peach Fruit

#### 3.4.1. Decay Incidence

**Figure 4:**
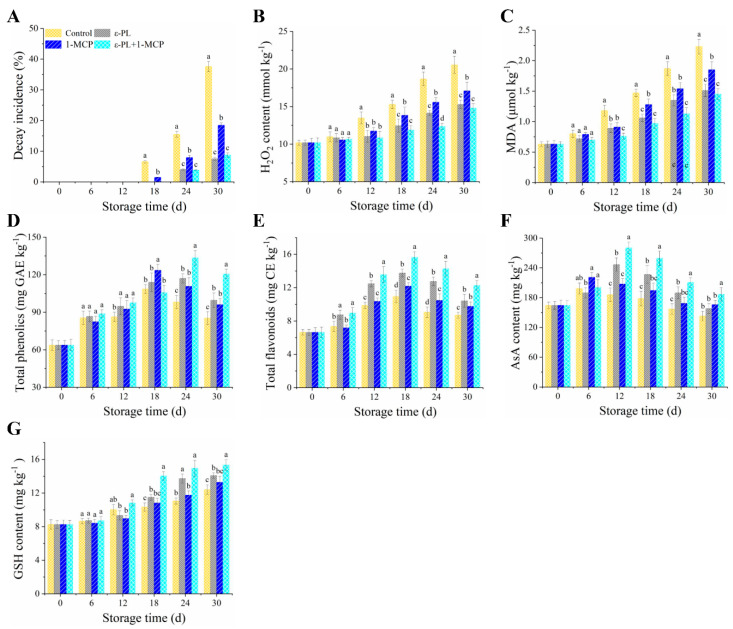
The variation in decay incidence (**A**), H_2_O_2_ (**B**), MDA (**C**), total phenolics (**D**), total flavonoids (**E**), AsA (**F**), and GSH (**G**) in flat peach fruit subjected to ε-PL, 1-MCP, and ε-PL+1-MCP treatments during a 30−day storage at 0 °C. Each bar represents mean ± standard deviations over three replications; distinctive letters denote statistical variances (*p* < 0.05) at individual intervals.

During storage, flat peach fruit manifested enhanced susceptibility to pathogenic infections. Implementations of ε-PL, 1-MCP, and the combined ε-PL+1-MCP markedly (*p* < 0.05) curtailed the spoilage rates over the storage duration (Figure 4A). Post 30 days of cold storage, ε-PL, 1-MCP, and the combined ε-PL+1-MCP treatments yielded decay rates significantly reduced to 79.96%, 50.88%, and 76.77%, respectively, when juxtaposed with controls. From days 24–30, the decay rates between the ε-PL and the combined ε-PL+1-MCP groups were statistically analogous.

#### 3.4.2. H_2_O_2_ and MDA Contents

Relative to controls, treatments with ε-PL, 1-MCP, and ε-PL+1-MCP on the 30th day led to a diminished H_2_O_2_ concentration, equating to 25.61%, 16.80%, and 28.04% of the control, in that order (Figure 4B). MDA levels in the peaches, irrespective of treatment, inclined, suggesting amplified oxidative cellular compromise as the storage interval extended (Figure 4C). By the 30th day, control samples exhibited MDA levels at 2.23 ± 0.12 μmol kg^−1^, markedly (*p* < 0.05) surpassing those in ε-PL, 1-MCP, and ε-PL+1-MCP treatments. However, ε-PL and the combined ε-PL+1-MCP efficaciously curbed MDA accumulation, registering 1.51 ± 0.10 μmol kg^−1^ and 1.45 ± 0.09 μmol kg^−1^, respectively.

#### 3.4.3. Total Phenolics, Total Flavonoids, AsA, and GSH Contents

A discernible amplification in total phenolic contents was noted in the peaches, succeeded by a minor decrement as storage persisted (Figure 4D). In relation to the untreated specimens, 30-day post-storage levels in peaches administered with ε-PL, 1-MCP, and the combined ε-PL+1-MCP surged by 16.78%, 12.63%, and 41.36%, correspondingly. From 24–30 d, the combined ε-PL+1-MCP exhibited a notably augmented phenolic content compared to either ε-PL or 1-MCP in isolation. Across the storage phase, ε-PL, 1-MCP, and ε-PL+1-MCP treatments bestowed peaches with superior total flavonoid concentrations relative to controls (Figure 4E). A zenith of 15.63 ± 0.68 mg kg^−1^ in total flavonoids was reached on day 18 in peaches treated with the combined ε-PL+1-MCP, overshadowing all alternative groups.

The AsA content in both the control and 1-MCP groups peaked on the 6th day of storage, registering 198.38 ± 11.35 mg kg^−1^ and 220.81 ± 9.52 mg kg^−1^, respectively (Figure 4F). On the 12th day, the ε-PL and ε-PL+1-MCP groups observed their highest AsA values, noting 246.57 ± 13.63 mg kg^−1^ and 280.32 ± 11.9 mg kg^−1^, respectively. Throughout the evaluation, flat peaches treated with ε-PL, 1-MCP, and ε-PL+1-MCP consistently presented elevated AsA contents in contrast to the control. Additionally, from the 18th–30th day, treatments of ε-PL, 1-MCP, and ε-PL+1-MCP notably enhanced the GSH content (Figure 4G). On the final day of storage, the ε-PL+1-MCP-treated peaches achieved a GSH content peak of 15.34 ± 0.62 mg kg^−1^, marking a 1.24-fold increase relative to the control.

### 3.5. APX, GR, POD, CAT, and SOD Activities

**Figure 5:**
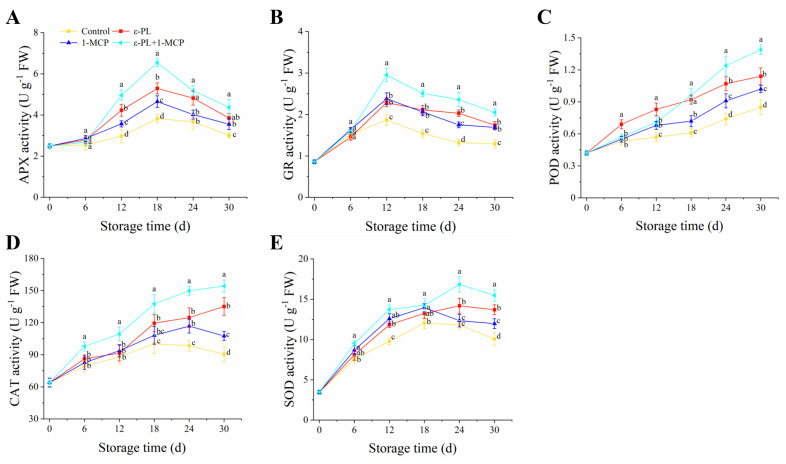
APX (**A**), GR (**B**), POD (**C**), CAT (**D**)and SOD (**E**) activities of flat peach fruit with ε-PL, 1-MCP, and ε-PL+1-MCP over a 30−day storage at 0 °C. Results are presented as mean ± standard deviations (*n* = 3), with distinct letters signifying notable treatment differences at each sampling time (*p* < 0.05).

Throughout the first 18 days, the APX activities in flat peaches undergoing ε-PL, 1-MCP, and ε-PL+1-MCP treatments displayed an initial rise, followed by a steady decline between the 18th and 30th days (Figure 5A). By the end of storage, treated peach fruits registered APX activities that were 27.91%, 17.94%, and 45.18% superior to the control. The GR activity in peach pulp treated with ε-PL, 1-MCP, and ε-PL+1-MCP was similar to the trends of APX activity (Figure 5B). From 12–30 d, ε-PL, 1-MCP and ε-PL+1-MCP applications enhanced the increase in GR activities. POD activities in flat peaches under different treatments increased with the extended storage (Figure 5C). Between days 12 and 30, compared to the control group, all three treatment groups resulted in an increase in POD activity. Dramatically, ε-PL+1-MCP showed significantly higher (*p* < 0.05) POD activity from 24–30 d when compared to ε-PL or 1-MCP alone. The CAT activity of the control group and 1-MCP group reached its maximum on the 18th and 24th days of storage, respectively. Both values were less than of the combined treatments (*p* < 0.05) (Figure 5D). Compared to control samples, the CAT activities in the peach treated with ε-PL, 1-MCP, and ε-PL+1-MCP were increased by 49.14%, 18.73%, and 70.27%, respectively, following 30 d of storage. The SOD activities of peach pulps displayed an initial increase followed by a subsequent decline (Figure 5E). Over days 24–30, both the ε-PL and ε-PL+1-MCP treatments substantially augmented SOD activities.

### 3.6. PAL, C4H, and 4CL Activities

**Figure 6:**
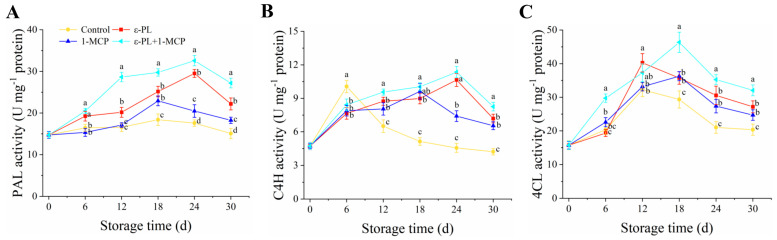
PAL (**A**), C4H (**B**), and 4CL (**C**) activities in flat peach post−treatment with ε-PL, 1-MCP, and ε-PL+1-MCP over a 30−day storage at 0 °C. Results denote mean ± standard deviations (*n* = 3), with distinct letters indicating statistically significant discrepancies among treatments for each time stamp (*p* < 0.05).

Throughout the storage period, PAL activity in flat peach tissues exhibited an initial rise, followed by a decline (Figure 6A). In the peach fruit treated with ε-PL+1-MCP, PAL activity was notably higher than other groups from 12–30 d (*p* < 0.05). The peak of PAL activity for flat peaches with ε-PL+1-MCP treatment occurred on the 24th day of storage, registering an increase of 1.85 times compared to the control. For all groups, the pulp C4H activity in flat peach rose initially, then dropped (Figure 6B). Although C4H activity in the control fruit was higher than in other groups on day 6 of storage, ε-PL, 1-MCP, and ε-PL+1-MCP groups enhanced C4H activity to varying degrees from 12–30 d. C4H activity in the peaches treated with ε-PL+1-MCP peaked at storage day 24, with a 2.49-fold increase compared to controls over the same period. In addition, ε-PL+1-MCP showed higher in C4H activity from 12 d to 30 d when compared to ε-PL or 1-MCP alone. The 4CL activity in the control peach raised from 0–12 d of storage, followed by a decline from 12 d to 30 d (Figure 6C). The 4CL activity in the peaches treated with ε-PL, 1-MCP, and ε-PL+1-MCP groups within the storage 18–30 d was significantly higher than the control group (*p* < 0.05).

### 3.7. Pearson’s Correlation Coefficient Analysis

**Figure 7:**
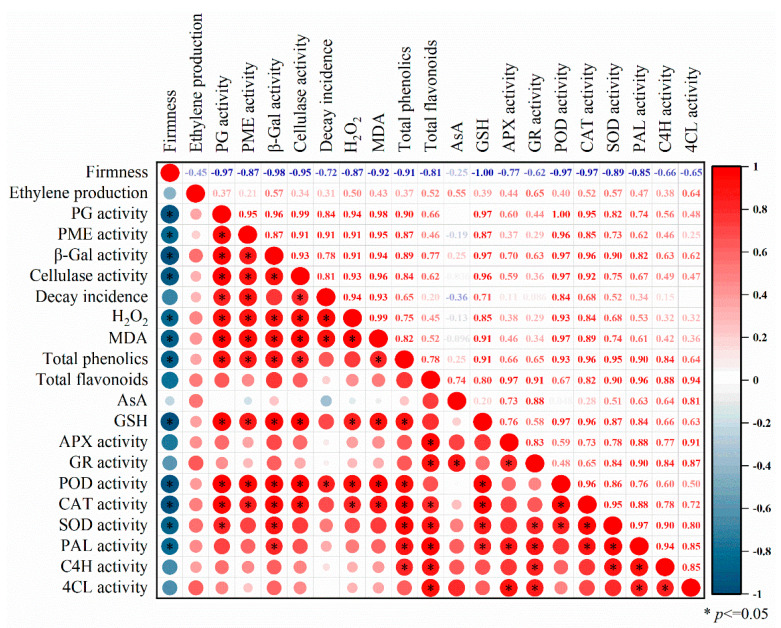
Correlation matrix between the determined parameters involved in quality attributes and related enzymes activity in flat peach fruit of ε-PL+1-MCP treatment during 30 d of storage. Statistically significant variances are marked by asterisks (* *p* ≤ 0.05).

Pearson’s correlation analysis was used to evaluate the potential relations between firmness and cell-wall-degrading enzymes as well as between decay incidence, reactive oxygen species, and phenylpropanoid metabolism indexes of peach fruit (Figure 7). During 0–30 d of storage, the declined peach pulp firmness in ε-PL+1-MCP treatment had a significantly negative correlation with the increased PG, PME, *β*-Gal, and cellulase activities (*p* ≤ 0.05). The decay rate shows a positive correlation trend with H_2_O_2_ and MDA, while H_2_O_2_ is positively correlated with MDA, GSH, POD, and CAT. In addition, total phenolics was positively correlated with the GSH, POD, CAT, SOD, PAL, and C4H, while total flavonoids was significantly positive correlated with the APX, GR, CAT, SOD, PAL, C4H, and 4CL activities.

## 4. Discussion

The softening of fruit is a major aspect that affects transportability, storage, and shelf life. One of the most direct characteristics of quality loss during storage is the fast decrease in peach fruit firmness. In this study, the firmness of peach fruit showed a significant downward trend during the storage cycle. However, 1-MCP treatment maintained the firmness of flat peaches in cold storage. Similar results have also been obtained in other fruit, such as cheri-moyas [41], nectarines [42], peaches [43], kiwifruits [44], plums [45], and apples [46]. As a natural, safe, and efficient food preservative, ε-PL treatment can improve the quality of fruit [22]. The result indicated that ε-PL application slightly retarded the flat peaches softening during storage. The ε-PL combined with 1-MCP showed similar effects to 1-MCP alone and delayed the fruit softening better than ε-PL alone. Agreeing with our result, Lin et al. [9] also found that the activities of cell-wall-degrading enzymes in 1-MCP-treated plums were obviously lower than those in control fruit, which decreased disassembly of cell wall polysaccharides and maintained firmness. 1-MCP and ε-PL+1-MCP decelerated the ethylene production, indicating that those groups effectively hindered ethylene action in the peach fruit [47]. 1-MCP can bind to ethylene receptors in the postharvest fruit due to a copper carbenoid intermediate forming in the cyclopropene-ring-opening reaction mechanism [48]. The copper carbenoid intermediate irreversibly reacts with amino acids of the protein domain in ethylene receptor to block the ethylene action. Hayama et al. [49] found that the combined treatment of aminoethoxyvinylglycine and 1-MCP reduced ethylene production and retarded melting-flesh peach softening. Additionally, the results from microstructure observation demonstrated that the softening of peach fruit was concomitant with disruption of the pulp cell tissue. ε-PL+1-MCP treatment retarded the degradation of cell tissue structure of peach fruit and maintained the tissue structure integrity, which was corroborated by previous findings on ethylene-treated peaches [50].

Fruit softening during ripening associates with the polysaccharide compositions degradation in cell wall, which is modulated by several enzymes such as PG, PME, *β*-GAL, and cellulase [31]. Specifically, PG facilitates the conversion of pectic acid into galacturonic acid by hydrolyzing the 1,4-α-D-galacturonic bond. PME, as a substrate for PG, can de-esterify methoxylated pectin and catalyze the galacturonic acid polymer decomposition to polygalacturonic acid [51]. In the current study, ε-PL, 1-MCP, and ε-PL+1-MCP treatments suppressed increases in PG and PME activities in flat peach. A previous study showed that a synergistic application of hot air and 1-MCP could suppress PG and PME activities in nectarines, thereby retarding fruit softening [52]. Moreover, *β*-Gal, a critical enzyme in pectin debranching, partakes in the hydrolysis of *β*-1, 4-galactan bonds. Moreover, *β*-Gal, as a critical enzyme of pectin-debranching, is involved in the hydrolysis of *β*-1, 4-galactan bonds. Cellulase can cause cellulose degradation in the cell wall [53]. During the entire storage period, ε-PL-, 1-MCP-, and ε-PL+1-MCP-treated peaches presented lower *β*-GAL and cellulase activities compared to the control fruit. Win et al. [46] found that 1-MCP suppressed *β*-galactosidase activity in apples, contributing to a delay in the degradation of pectin polysaccharides. Agreeing with our result, Xiong et al. [54] found that 1-MCP-treated kiwifruit mainly delays fruit softening by inhibiting cellulase activity and cell wall decomposition. In this study, the decreased peach pulp firmness in ε-PL+1-MCP treatment had a significantly negative correlation with the increased PG, PME, *β*-Gal, and cellulase activities (*p* ≤ 0.05). Our results showed that ε-PL+1-MCP was effective in delaying flat peach softening by suppressing the above enzymatic activities. Furthermore, the results from ultrastructural observation again provided direct evidence that flat peach fruit softening was accompanied by disruption of the cell wall structure. Importantly, ε-PL+1-MCP treatment delayed cell wall degradation, and this served to maintain the integrity of the cell wall structure and further delay the softening process of cold-stored peach fruit, which was in line with previous reports demonstrated in nectarine treated with hot air and 1-MCP [52] and apricot [55] treated with 1-MCP.

Enormous economic losses in the flat peaches during storage, transportation, and retail are caused by pathogen infection [56]. ε-PL has been previously applied to enhance fruit disease resistance in peaches [51], longans [57], tomatoes, apples, and jujubes [58], thereby reducing postharvest loss. In this study, ε-PL and ε-PL+1-MCP treatments inhibited the decay of the peach fruit during postharvest storage, which was attributed to effective antimicrobial activity against microorganisms of ε-PL’. Studies have found that both reactive oxygen species (ROS) and phenylpropanoid pathway metabolisms are involved in inducing disease resistance in fruit [59]. Antioxidant enzymes and non-enzymatic antioxidants, including reduced GSH and AsA, regulate ROS level in plants [60]. Oxygen is reduced to superoxide anion (O_2_^•−^) under the action of nicotinamide adenine dinucleotide phosphate oxidase, which is disproportionated to H_2_O_2_ by the SOD in the plant cells [59]. As antioxidant enzymes, POD and CAT can catalyze H_2_O_2_ into H_2_O and O_2_ to prevent excessive ROS accumulation in plant cells, while APX and GR also participate in ROS elimination through the AsA–GSH cycle. In this work, ε-PL application raised AsA and GSH contents and the activities of APX, GR, POD, CAT, and SOD and decreased H_2_O_2_ content in flat peaches during storage, strengthening resistance against pathogens. A study revealed that ε-PL upregulated the relative gene expression of *MdAPX*, *MdPOD*, *MdCAT*, and *MdSOD* to control *Alternaria* rot of postharvest apples [54]. The flat peach fruit with 1-MCP had higher decay rate compared with the ε-PL-treated fruit. Importantly, ε-PL and ε-PL+1-MCP applications were similar, and both reduced in the rot of postharvest peaches. The decay incidence in ε-PL+1-MCP was positively correlated with H*_2_*O_2_ and MDA. These results showed that ε-PL combined with 1-MCP inhibited H_2_O_2_ and MDA accumulation and induced fruit disease resistance.

Secondary metabolites generated from the phenylpropanoid pathway maintain integrity of plant cell walls and protect plants against pathogenic invasion [56]. PAL, C4H, and 4CL play a significant role in the biosynthesis of secondary metabolites. ε-PL+1-MCP increased the activities of PAL, C4H, and 4CL, leading to an increase in flavonoids and total phenolic contents, thus improving resistance against pathogenic bacteria in peach fruit. This is because the flavonoids and phenolic compounds have antiviral and antimicrobial activity [59]. Our findings indicated that the total phenolics in ε-PL+1-MCP was positively correlated with the PAL, and C4H activities, while the total flavonoids had significantly positive correlated with the PAL, C4H, and 4CL activities. Fan et al. [24] reported similar results that ultrasound combined with ε-PL treatment maintained a high total phenolics content to effectively inhibit microorganism growth. ε-PL reduced disease development via a direct effect on activities of PAL, C4H, and 4CL in longan fruits [57]. These results indicate that ε-PL+1-MCP effectively reduced fruit decay by maintaining cell wall integrity; enhancing the activities of PAL, C4H, and 4CL enzymes; and increasing the levels of total phenolic and flavonoids in peaches throughout the storage period.

## 5. Conclusions

In summary, the combination of 1 µL L^−1^ 1-MCP fumigation and 300 mg L^−1^ ε-PL not only delayed the softening but also maintained a lower decay rate of flat peaches stored at 0 °C for 30 days after harvest. The composite treatment has a good effect on maintaining fruit firmness due to the inhibited PG, PME, *β*-Gal, and cellulase activities retarding the degradation of cell wall structure in peaches. The reactive oxygen species (ROS) and phenylpropanoid pathway metabolisms were regulated by ε-PL and 1-MCP. Thus, the postharvest decay of peaches was reduced. These results imply that a synergistic application of ε-PL and 1-MCP presents a promising strategy for improving the quality of peach fruit.

## Data Availability

The datasets generated for this study are available on request to the corresponding author.
